# Soil-transmitted helminths detected from environmental samples in a campus of southern Brazil

**DOI:** 10.1016/j.soh.2023.100016

**Published:** 2023-05-10

**Authors:** Marina Ziliotto, Joel Henrique Ellwanger, José Artur Bogo Chies

**Affiliations:** Laboratory of Immunobiology and Immunogenetics, Department of Genetics, Postgraduate Program in Genetics and Molecular Biology (PPGBM), Universidade Federal do Rio Grande do Sul (UFRGS), Porto Alegre, Rio Grande do Sul, Brazil

**Keywords:** Environment, Hookworm, Geohelminths, Roundworm, Soil-transmitted helminths, Whipworm

## Abstract

Soil harbours enormous biodiversity, essential for maintaining environmental and human health. However, soil can also be a reservoir of various parasitic pathogens, such as soil-transmitted helminths (STH). We evaluated the presence of STH (e.g., hookworms, roundworms and whipworms) in soil samples collected at twenty points within the perimeter of *Campus do Vale* (a university campus belonging to the Federal University of Rio Grande do Sul - UFRGS), during 2022 winter season. Considering the One Health perspective, human, animal and environment-related data from each sampling point were collected. All soil samples showed nematode larvae, representing natural components of soil biodiversity. Considering STH eggs, 35% (*n* = 7) of soil samples showed hookworm eggs (e.g., from *Necator americanus* or *Ancylostoma duodenale*), 10% (*n* = 2) showed roundworm (*Ascaris lumbricoides*) eggs, and 5% (*n* = 1) showed whipworm (*Trichuris trichiura*-like) eggs. Of note, 10% of the sampling points showed the presence of rhabditiform hookworm larvae, 5% showed *Strongyloides stercoralis* rhabditiform larvae and 5% had the presence of filariform hookworm larvae, indicating a risk of human percutaneous infection. The significant people circulation in *Campus do Vale*, in association with other environment-related factors, help to explain the prevalence of STH observed in this study.

## Highlights

• The prevalence of soil-transmitted helminths (STH) in southern Brazil is a neglected issue
• This study investigated STH distribution in soil samples based on the One Health perspective
• Eggs of hookworms, roundworms and whipworms were found
• Larvae of hookworms and *Strongyloides stercoralis* were observed
• Human, animal and environmental factors help to explain the STH prevalence found in this study

## 1 Introduction

Helminths are the most common human pathogenic parasites in developing countries. Helminth infections are major neglected tropical diseases considering disability-adjusted life years and other criteria [1]. Soil-transmitted helminths (STH) are enteric parasites with a biological cycle that includes an obligatory developmental stage in the soil, being capable of infecting humans and non-human animals through contact with contaminated soil. Roundworms (*Ascaris lumbricoides*), whipworms (*Trichuris trichiura*) and hookworms (*Ancylostoma duodenale* and *Necator americanus*) are considered the main human STH in different world regions [2].

Human infection by whipworms and roundworms usually occurs through the ingestion of mature eggs after contact with egg-contaminated soil or food. Third-stage hookworm larvae (L3 or filariform) infect humans through percutaneous invasion (*N. americanus* and *A. duodenale*). Infection by *A. duodenale* can also occur by accidental oral ingestion, potentially resulting in Wakana syndrome [2,3]. *Strongyloides stercoralis* is also considered a member of the STH group; however, many health agencies do not include this parasite in their disease control programs. Therefore, *S. stercoralis* is often not considered in STH studies [4]. The population infected by *Strongyloides* is estimated at 30–100 million people worldwide [2]. Free-living *Strongyloides* filariform larvae found in the soil can infect humans through direct contact [5]. The soil can therefore serve as an ‘intermediate host’ during STH infection. STH eggs and larvae can develop and become infective in soils with proper conditions for the development and protection of the parasites [6].

Some hookworm species are classified as zoonotic parasites (e.g., *Ancylostoma brazilense*, *Ancylostoma caninum*) having animals (e.g., dogs, cats) as definitive hosts, but accidentally parasitizing humans. This zoonotic infection causes cutaneous larva migrans, an inflammatory disease triggered by the larvae migration through the human skin [7,8].

STH infections are important causes of impairment of physical growth and intellectual development in children, and can also trigger various health issues in adults, such as malnutrition, iron deficiency anemia and intestinal problems [3,9,10]. Eggs and larvae of STH are very adapted to tropical and subtropical climates, and both mono- and co-infection by STH are of great public health significance to low- and middle-income countries in Africa, Asia and Latin America, including Brazil [2,3,11].

The complex and porous structure of the soil harbours an enormous quantity and variety of animal species and microorganisms. The soil biodiversity varies depending on the content of organic material, chemicals, food supplies, water and sanitary condition of the environment. The soil biodiversity is also affected by anthropogenic activities, such as urbanization, agriculture and other types of land-use changes. Considering the enormous diversity of archaea, bacteria, protists, tardigrades, rotifers, nematodes, acari, collembolans, worms, macroarthropods and burrowing mammals found in soil, the current knowledge about soil biodiversity is limited. This occurs because (I) the identification of soil-associated species is difficult, (II) the small size of the organisms, (III) the lack of qualified professionals, among other reasons. This lack of knowledge refers to both organisms beneficial to humans and pathogenic species [12,13].

Nematodes comprise an important part of soil biodiversity, being considered good indicators of environmental quality. Soil nematodes include parasites that can infect plants, animals and humans (i.e. STH). The distribution of soil nematodes is affected by solar incidence, vegetation and chemical and physical characteristics of the soil [13]. STH are observed in several Brazilian regions and their prevalence is associated with the socioeconomic conditions of the population. Lack of access to safe water, sanitation and hygiene (WASH) and precarious housing conditions are important factors for environmental contamination and human infection by STH [14,15].

Katz [16] performed a national survey concerning STH prevalence in the Brazilian population, covering all Brazilian states. The Rio Grande do Sul state had a limited adherence to the survey (36.9%), compromising the reliability of the results (adherence to the survey ranged from 60% to 100% among the other states). Therefore, new regional studies are needed to analyze and update STH data for Rio Grande do Sul state, especially considering Porto Alegre city, the most populous city of the state. Moreover, Porto Alegre lost two places in Brazil's “sanitation ranking”, now occupying position number 42. This unwelcome result may affect the occurrence of STH in the city. Of note, the sewage system network reaches only 91.3% of the Porto Alegre population, and the city only treats 82% of all the collected sewage ([17]; based on 2020 data). The lack of proper treatment of sewage produced in the city causes the dumping of organic carbon into Lake Guaíba, a water body of great environmental, economic and historical-cultural importance for the state, thus increasing levels of metals and phosphorus in the lake [18]. Taken together, these issues collaborate to increase the risks of STH infection for the population living in the Porto Alegre region.

Other factors make Porto Alegre an interesting city for parasitological studies. Porto Alegre is in an ecotone region. The diversity of geomorphological and biological structures observed in the transition between two biomes generates a mosaic of hills and plains covered by forests and grasslands [19]. *Campus do Vale* belongs to the Federal University of Rio Grande do Sul, one of the largest universities in Brazil, being located between the biomes Atlantic Forest (*Mata Atlântica*) and Pampa. *Campus do Vale* harbours the highest hill in the Porto Alegre city, called *Morro Santana*, which is an important and protected “green area”. This ecological reserve is home to an enormous diversity of mammals [19] and about a quarter of the estimated grass diversity of the state [20]. Also, around 60 bird species were observed within *Campus do Vale* [21]. Many students, employees and members of the general population frequent *Campus do Vale* daily, and due to its biodiverse landscape, human interaction with insects, birds, primates and reptiles is common (authors' observation). Moreover, the presence of domestic animals, mainly dogs, circulating among people is a common observation in *Campus do Vale*. This occurs due to residential neighborhoods located very close to the campus associated with the university community's habit of providing shelter and food for dogs.

Considering the scarcity of data concerning STH in the Porto Alegre region combined with the interesting ecological characteristics of *Campus do Vale*, the objective of this study was to evaluate the presence of STH eggs and larvae in soil samples at twenty sampling points distributed throughout the *Campus do Vale* (UFRGS), Porto Alegre, southern Brazil. Environmental, animal and human information and data were collected from each sampling site to help explain the results. This is a study developed under the One Health perspective [22,23] since it evaluated a complex public health problem considering human, animal and environmental aspects. Finally, we highlight that this is a pilot study for another larger study involving different sampling points located in the Porto Alegre region.

## 2 Materials and methods

### 2.1 Ethical statement

Soil sample collections were authorized by the *Sistema de Autorização e Informação em Biodiversidade* - SISBIO (*Instituto Chico Mendes de Conservação da Biodiversidade* - ICMBio, *Ministério do Meio Ambiente*, Brazil): SISBIO No. 82718–1. This study was also authorized by the Environment Department of UFRGS (DMALIC/SUINFRA - UFRGS). Biological samples collected during this study were also registered in the *Sistema Nacional de Gestão do Patrimônio Genético e do Conhecimento Tradicional Associado* - SisGen (*Ministério do Meio Ambiente*, Brazil): registration codes ADC0B9C and A314A58.

### 2.2 Study area

**Fig. 1:**
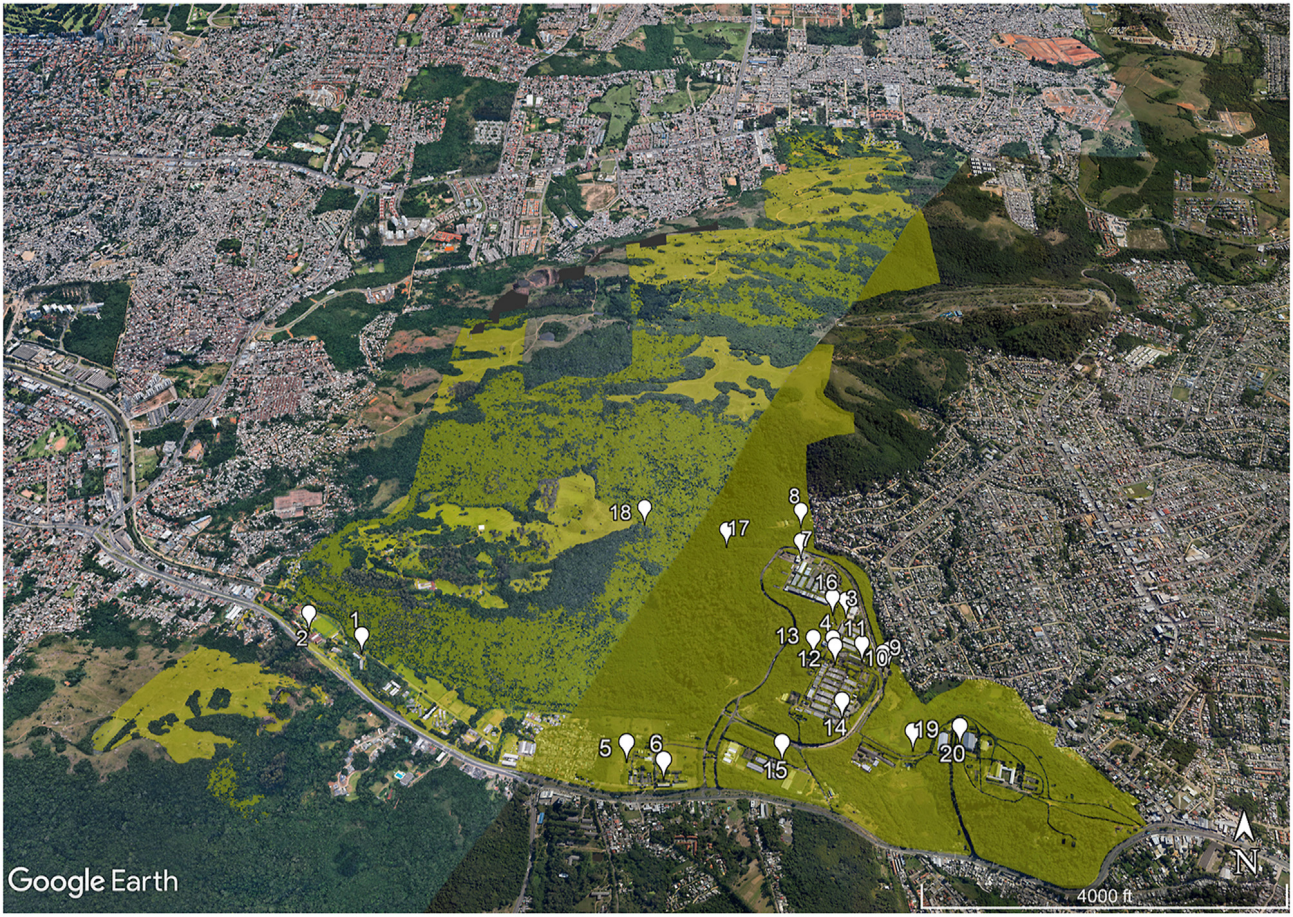
**Sampling points distributed throughout *Campus do Vale* - UFRGS.** Each number represents a sampling point. The name and characteristics of each sampling point are detailed in Table 1.

Soil samples were collected at twenty points of *Campus do Vale* (Fig 1 and Table 1), a university campus belonging to UFRGS and localized in Porto Alegre city, the capital of Rio Grande do Sul state, extreme south of Brazil. Porto Alegre has a territorial area of 495,390 km^2^ [24] and an estimated population of 1,492,530 people [25]. Porto Alegre is an ecotone zone, composed of Atlantic Forest and Pampa (grasslands) biomes. The vegetation cover of Porto Alegre was heavily modified due to urbanization, agriculture and mining activities, among other types of anthropogenic land-use changes. Only 24.1% of the original vegetation cover remains in the region: 10.2% of grasslands and 13.9% of forest cover [26,27].

**Table 1:** Geographic location, soil characteristics, vegetation and climatic conditions of each point recorded during the sampling.

Sampling point	Popular name^a^ (translated from Portuguese)	Latitude	Longitude	Temp.	Climate condition	Soil characteristic	Vegetation
Point 1	University Restaurant no. 4, Agronomy	−30.071308	−51.138732	11 °C	Cloudy	Clayey	Ornamental
Point 2	Phylogenetic Garden, Agronomy	−30.070267	−51.140178	10 °C	Cloudy	Organic	Ornamental
Point 3	Department of Genetics	−30.069913	−51.119114	10 °C	Sunny	Organic	Ornamental
Point 4	Campus do Vale Center	−30.071547	−51.119821	10 °C	Sunny	Organic/Sandy	Ornamental
Point 5	Waste Center, Veterinary Medicine	−30.075629	−51.128026	14 °C	Sunny	Organic	Degraded natural grassland/shrub
Point 6	Academic Directory, Veterinary Medicine	−30.076296	−51.126637	14 °C	Sunny	Organic	Ornamental
Point 7	University Restaurant no. 6, Campus do Vale	−30.067304	−51.120744	13 °C	Sunny	Organic	Ornamental
Point 8	Patas Dadas	−30.065938	−51.120634	12 °C	Sunny	Organic	Degraded/artificial/reforested arboreal forest
Point 9	Neighborhood Entrance	−30.071983	−51.117632	13 °C	Rainy	Clayey	Degraded natural grassland/shrub
Point 10	Bus station	−30.072135	−51.117889	13 °C	Rainy	Organic	Ornamental
Point 11	Language and Arts Building	−30.071798	−51.118693	13 °C	Rainy	Organic	Ornamental
Point 12	Square between Language and Chemistry courses	−30.071890	−51.119771	14 °C	Rainy	Organic	Ornamental
Point 13	University Restaurant no. 3, Campus do Vale	−30.071513	−51.120597	14 °C	Rainy	Sandy	Degraded/artificial/reforested arboreal forest
Point 14	Microscopy Center	−30.074131	−51.119747	14 °C	Rainy	Organic	Ornamental
Point 15	Bus station, Aplicação school	−30.075950	−51.121351	15 °C	Rainy	Sandy	Ornamental
Point 16	Stairway	−30.069863	−51.119667	15 °C	Rainy	Organic	Degraded/artificial/reforested arboreal forest
Point 17	Santana Hill – entrance	−30.066905	−51.123875	15 °C	Sunny	Organic	Preserved arboreal forest
Point 18	Santana Hill–center	−30.066087	−51.127359	15 °C	Sunny	Organic	Preserved arboreal forest
Point 19	Dam Lake	−30.075259	−51.117172	11 °C	Sunny	Organic	Degraded/artificial/reforested arboreal forest
Point 20	Hydraulic Research Institute	−30.075140	−51.115393	11 °C	Sunny	Organic	Degraded/artificial/reforested arboreal forest

a Names commonly used by the University's community. Temp. = temperature (average).

*Campus do Vale* has an extension of ∼700 ha, with an estimated community of 30,000 people [28]. The campus includes more than 30 undergraduate courses, two university restaurants and six research centers. Some university units provide direct services to the community, such as the University's Veterinary Hospital and the Institute of Hydraulic Research (*Instituto de Pesquisas Hidráulicas* - IPH). Also, a dog shelter managed by an animal protection NGO operated until recently within the *Campus do Vale* (the NGO's activities ceased in March 2023). *Campus do Vale* encompasses part of *Dilúvio* Stream and the *Morro Santana* Ecological Reserve, which covers an area of 3,500,000 m^2^. This area also includes a region once occupied as a farmland, which was posteriorly acquired by the Brazilian government. *Campus do Vale* is an ecological reserve composed of natural landscapes with varied levels of regeneration. This ecological reserve exhibits a vegetation profile that includes rupestrian fields and regenerated subxerophytic and mesophyll forests [26].

### 2.3 Sampling method and evaluation of soil samples

Soil samples were collected and processed according to the Rugai's method [29] adapted for soil samples [30], during 2022 winter season. From each sampling point, we collected approximately 100 g of topsoil (up to 10 cm deep) using a clean garden shovel and sterile plastic tubes. The sampling points were selected with the objective of sampling areas of *Campus do Vale* with different landscape characteristics concerning vegetation, presence of people and animals, and types of building (Table 1). The soil samples were placed into a polystyrene box and transported to the laboratory (Laboratory of Immunobiology and Immunogenetics - UFRGS). All samples were analyzed immediately after collection.

Each 100 g of freshly extracted soil were placed in four-layer packs of bandage gauze. The packs containing the soil samples were then placed in glass sedimentation cones filled with 45 °C water. The water temperature allows the capture of helminth larvae due to their thermotropism and hydrotropism. The eggs of the parasites sediment by gravity, and larger soil components remain trapped in the four-layer packs of bandage gauze. After 1-h sedimentation, packs were removed from sedimentation cones and the material continued to settle for an additional 1 h. Afterwards, the sedimented material was removed from the bottom of the sedimentation cones with disposable Pasteur pipettes and transferred to microcentrifuge tubes, then centrifuged at 2000 rpm (380 G-force) for 2 min. Finally, an aliquot of the centrifuged material was placed under a microscope slide, stained with 2% Lugol, and covered with a coverslip. The slides were analyzed under the microscope by two trained microscopists under 100× and 400× magnification. The above-mentioned Rugai's method adapted for soil samples was based on Carvalho et al. [30], with minor adaptations. Studies performed by González y Cáceres et al. [31] and Carvalho et al. [32] were also considered during methodology standardization. This method allows the recovery of the following parasitic structures: eggs of *A. lumbricoides*, hookworms, *Taenia* sp., *Trichirus trichiura* and *Toxocara canis*, larvae of *S. stercoralis* and hookworms, and cysts of *Giardia lamblia*, *Entamoeba coli* and *E. histolytica* [30]. For this study, parasite cysts were not considered. Two slides were analyzed for each sampling points (one slide per microscopists; 20 sampling points), totaling 40 slides in this study. The morphological identification of the parasites was based on the descriptions and recommendations of Neves [33], De Carli [34], Mariano et al. [35], and CDC [36].

### 2.4 Collection of environment-related data

A form for the assessment of socio-environmental data and anthropic activity (available in Brazilian Portuguese as Supplementary Material) was filled at each sampling point. In this form, we recorded data concerning biotic and abiotic aspects that could influence the presence or absence of soil parasites, such as conditions of environmental sanitation, disposal of domestic and industrial waste, presence of regular housing, circulation and signs of animals and humans, among other environmental characteristics.

## 3 Results

Table 1 shows detailed data concerning weather conditions, temperature, soil characteristic and vegetation of each sampling point. Soil samplings were performed during the winter season and due to this reason the temperatures at sampling days were mild (12.8 °C average) with half of sampling days showing rainy or cloudy conditions. Eleven sampling points (55%) showed ornamental vegetation; and organic soil was observed in most of the sampling points (*n* = 16, 80%).

Table 2 shows results from environment-related data recorded at each sampling point. Most of the sites showed regular human dwellings/buildings and proper environmental sanitation (*n* = 15, 75%; for both criteria). However, inappropriate domestic solid waste disposal was observed in 15 (75%) sampling points. Due to the presence of *Dilúvio* Stream, which passes through the *Campus do Vale*, the disposal of both domestic and industrial sewage was observed at sampling point 19. Near the University Restaurant (sampling point 7), we also observed problems in the building plumbing, with domestic sewage partially licking into the environment. Domestic animals (dogs) were observed in only two sampling points. Observation or indicatives of synanthropic animals (especially pigeons and rodents) were recorded in 6 (30%) sampling points. The main indicator of synanthropic animals were rodent traps spread across the campus. Farm animals were observed only at the University's veterinary area (sampling point 5). Intense or frequent human presence was observed in 13 (65%) sampling points (Table 2).

**Table 2:** Environment-related characteristics of each sampling point.

Sampling point	Human dwellings/buildings	Noise pollution	Environmental sanitation^a^	Domestic sewage disposal	Artificial mosquito larvae breeding sites	Domestic solid waste	Industrial/biological sewage disposal	Expected human circulation	Domestic animals (sightings or indications)	Synanthropic animals (sightings or indications)	Farm animals (sightings or indications)
1	Regular	Absent/light sounds	Proper	Absent	Present	Present	Absent	Frequent	Present: dog	Absent	Absent
2	Regular	Present	Proper	Absent	Absent	Present	Absent	Frequent	Absent	Absent	Absent
3	Regular	Present	Proper	Absent	Absent	Present	Absent	Frequent	Absent	Absent	Absent
4	Regular	Present	Proper	Absent	Absent	Absent	Absent	Frequent	Absent	Absent	Absent
5	Regular	Absent/light sounds	Proper	Absent	Present	Present	Absent	Frequent	Absent	Present: pigeon	Present: cattle, horse
6	Regular	Present	Proper	Absent	Absent	Present	Absent	Frequent	Absent	Absent	Absent
7	Regular	Present	Proper	Present	Absent	Present	Absent	Intense	Absent	Present: pigeon	Absent
8	Regular	Absent/light sounds	Proper	Absent	Absent	Present	Absent	Infrequent	Present: dog	Present: rodent	Absent
9	Regular	Present	Proper	Absent	Absent	Present	Absent	Frequent	Absent	Present: pigeon	Absent
10	Absent	Intense	Not necessary	Absent	Absent	Present	Absent	Intense	Absent	Absent	Absent
11	Regular	Present	Proper	Absent	Absent	Present	Absent	Intense	Absent	Absent	Absent
12	Regular	Present	Proper	Absent	Absent	Absent	Absent	Intense	Absent	Absent	Absent
13	Regular	Intense	Proper	Absent	Absent	Present	Absent	Intense	Absent	Present: rodent	Absent
14	Regular	Absent/light sounds	Proper	Absent	Absent	Absent	Absent	Not frequent	Absent	Absent	Absent
15	Regular	Absent/light sounds	Proper	Absent	Absent	Present	Absent	Not frequent	Absent	Absent	Absent
16	Absent	Absent/light sounds	Not necessary	Absent	Absent	Present	Absent	Frequent	Absent	Absent	Absent
17	Absent	Absent/light sounds	Not necessary	Absent	Absent	Present	Absent	Not frequent	Absent	Absent	Absent
18	Absent	Absent/light sounds	Not necessary	Absent	Absent	Absent	Absent	Not frequent	Absent	Absent	Absent
19	Absent	Present	Inappropriate	Present	Present	Present	Present	Not frequent	Absent	Present: wild rodents, urban birds	Absent
20	Regular	Absent/light sounds	Proper	Absent	Absent	Absent	Absent	Not frequent	Absent	Absent	Absent

a Environmental sanitation: Presence of treated water and sewage collection systems. Domestic solid waste (presence/absence) was considered as a separate category in this article.

Table 3 details the results of parasitological analyses for each sampling point. Microscopic nematode larvae were observed in all sampling points, indicating that the parasitological technique used in this study was effective in the recovery of larvae present in the soil. However, we stress that most of the observed larvae are not pathogenic for humans or animals but are part of the normal (healthy) soil biodiversity. Considering pathogenic larvae, rhabditiform hookworm larvae were observed in sampling points 4 and 5. Filariform (infective) hookworm larvae were observed only at sampling point 4. *S. stercoralis* rhabditiform larvae were observed at sampling point 3 (Table 3).

**Table 3:** Parasite larvae and eggs observed at each sampling point.

Larvae and eggs identified	Sampling points																			
	1	2	3	4	5	6	7	8	9	10	11	12	13	14	15	16	17	18	19	20
Microscopic nematode larvae	X	X	X	X	X	X	X	X	X	X	X	X	X	X	X	X	X	X	X	X
Hookworm (filariform) larvae				X																
Hookworm (rhabditiform) larvae				X	X															
*Strongyloides stercoralis* (rhabditiform) larvae			X																	
Hookworm eggs	X			X				X	X		X			X						X
*Ascaris lumbricoides* eggs										X						X				
*Trichuris trichiura*-like^a^ eggs											X									

Each ‘X’ in the sampling point table cells indicates the presence of at least one of the parasitic structures (larvae or eggs) indicated in the column on the left. *Taenia* sp. and *Toxocara canis* eggs are not mentioned in the table because they were not observed in the analyses.

a Suggestive of *Trichuris trichiura* based on morphology.

Considering parasite eggs, hookworm eggs were found in 7 sampling points (35%), followed by *A. lumbricoides* eggs, observed in 2 sampling points (10%). *Trichuris trichiura*-like eggs were observed only at sampling point 11 (Table 3), a place of intense human presence (Table 2). *Taenia* sp. and *T. canis* eggs were not observed at any sampling point.

## 4 Discussion

Nematodes are one of the most diverse and widespread animal groups on the planet, participating in different levels of the soil food webs [37], and representing a significant part of the soil mass. Although some nematode species are parasitic on plants and animals, most species have a critical participation in the maintenance of soil health, thus providing food, increasing air and water quality, and regulating soil-related ecosystem services [13,38,39]. In regions constituted by grasslands, such as the Pampa biome, soil invertebrates contribute to plant species diversity and ecological succession [40]. Maintaining soil quality and nematode diversity are therefore critical for environmental and human health [12]. In this study, all soil samples showed an abundance of microscopic nematodes, indicating a rich soil biodiversity. This finding is probably due, at least in part, to the organic conditions of the soil found in most of the samples analyzed. Of note, morphological differentiation of pathogenic parasites (i.e., STH) from non-pathogenic nematodes in soil samples must be performed carefully due to the great abundance and diversity of nematodes found in soil. In the context of human parasitology, microscopists used to evaluate stool samples should be aware concerning the peculiarities and biodiversity of soil when analyzing STH in environmental samples. This careful approach is necessary in order to avoid misinterpretation of the presence of non-pathogenic nematodes, natural components of the soil biodiversity. Nevertheless, although most of the nematodes observed in the study were non-pathogenic components of soil, a small portion of the nematode larvae were part of the human pathogenic STH group.

STH are distributed throughout the Brazilian territory [16] and their prevalence is associated with socioeconomic conditions since lack of environmental sanitation, poor hygienic conditions, ingestion of non-potable water, walking barefoot and consumption of contaminated food are some factors that favor the occurrence of STH eggs and larvae in the soil and, consequently, human infection [8]. Adult stages of major STH inhabit the human gastrointestinal tract, where they can stay for months and even years. Hookworms and *Ascaris* sp. are usually found in the small intestine, whereas *T. trichuria* are found in the cecum, colon and rectum, depending on infection level [9]. Clinical aspects and morbidity are commonly associated with infection intensity [41], and STH eggs are passed through the feces to the external environment [2,42]. Eggs and larvae of STH can thrive in warm and moist soil for several months [2]. Hookworm larvae are free living organisms in the soil. The rhabditiform larva is the first larval stage, and it becomes infective when it reaches its third (filariform) larval stage [34].

In this study, we observed in the soil samples larvae of hookworms and *S. stercoralis*, eggs of *A. lumbricoides*, and suggestive eggs of *T. trichiura*. The presence of infective larvae demonstrates that the studied environment is indeed conducive to the survival and development of STH, even during the winter season, representing a risk for human infection. Our results are similar to other studies carried out in schools and university campi localized in southern Brazil (reviewed by Ziliotto et al. [43]), despite indicating that *Campus do Vale* is a less contaminated environment than public urban areas in Porto Alegre. In fact, a previous study performed by Vargas et al. [44] analyzed the presence of different parasitic structures in public squares and parks of Porto Alegre city; and 100% of the analyzed places were contaminated with some parasite [44].

The intense presence of people in *Campus do Vale* (∼30,000 individuals) [28] helps to explain the occurrence of STH in the study area, even with proper environmental sanitation conditions, as observed in most sampling points. *Campus do Vale* is frequented by many professors, students, employees and members of the general community, including children, once UFRGS provides a variety of services to the community (e.g., veterinary services, language courses, food services), which facilitates the direct and indirect deposition of STH eggs in soil of the study area. Of note, a residential neighborhood with precarious housing conditions is located right next to the *Campus do Vale*, which also helps to explain the presence of STH in the area even under adequate sanitation conditions. It is essential to stress that parasite eggs can be transported between different locations by domestic animals, the soles of shoes, and attached to invertebrates, among other ways [[45], [46], [47]]. Public transport can also contribute to the dissemination of eggs, larvae and cysts of intestinal parasites since parasitic structures can be attached and transported on the buses’ surface, for example [48]. Our study also highlighted issues that need to be improved in *Campus do Vale*, such as the disposal of waste observed at sampling point 19 (Dam Lake) and the frequent presence of domestic waste scattered throughout the campus. In brief, the soil of *Campus do Vale* is contaminated with STH and may pose a risk of human contamination, similarly to the risks offered by soils from parks and public squares. However, it is important to emphasize that this contamination risk depends on inadequate hygiene practices that lead to the accidental ingestion of soil and associated STH eggs, such as not washing hands after handling soil.

No *Taenia* sp. eggs were observed in the soil samples, which was an expected result. Although veterinary data confirm the circulation of *T. solium* and *T. saginata* in livestock from different regions of Rio Grande do Sul [49,50], both taeniasis and human cysticercosis are not major problems in the state. Although epidemiological data on human taeniasis and cysticercosis in Rio Grande Sul are scarce, the presence of *Taenia* eggs in soil samples is not common in public areas in Brazil. Considering seven Brazilian studies reviewed by Araújo et al. [51], only one study from the Northeast Region [52] reported *Teania* eggs in soil samples.

Furthermore, no *T. canis* eggs were observed in the soil samples in this study. Unlike *Taenia* sp., this was a surprising result since *T. canis* eggs were quite common in other similar studies (reviewed by Ziliotto et al. [43]). *T. canis* is a zoonotic parasite associated with the presence of dog feces [8]. Although signs of domestic animals were seen with some frequency in our study, the incidence of these animals was lower than expected (compared to authors’ observations/experience from previous years). This is potentially due to the drop in the circulation of people at the university during the COVID pandemic (and consequent decrease in food availability) and improvements in the dog shelter present at *Campus do Vale* before COVID pandemic. However, the absence of *T. canis* eggs in our samples can also be ascertained to methodological limitations. Although the morphological analysis based on microscopy is classically considered the gold standard in parasitology studies, the particular method we used may not be as accurate as molecular methods for *T. canis* eggs, representing a limitation of this study. As mentioned earlier, this is a pilot study that will serve as the basis for a larger study to be carried out with soil samples from various regions of Porto Alegre. For this new study, we therefore will include molecular analyses for the detection of *T. canis* DNA in the samples, along with the microscopy analyses.

Finally, the analysis of soil samples is an important approach for preventing possible parasite-related infection cases, as it suggests the infection risk to which individuals or populations are exposed to. Also, it contributes to targeting parasite infection control programs to areas with important levels of parasite contamination [53]. Considering the importance of medical and socioeconomic effects of neglected tropical diseases on Brazil population [54], limited information regarding STH infections are available for Rio Grande do Sul state. This information is essential for control and prevention programs focused on southern Brazil [43]. In this context, this study provides new and relevant data concerning environmental contamination by STH in Porto Alegre city.

## 5 Conclusions

This study reports the occurrence of larvae of hookworms and *S. stercoralis*, eggs of *A. lumbricoides*, and suggestive eggs of *T. trichiura* in soil samples of *Campus do Vale*, Porto Alegre, Brazil. The intense presence of people in the study area in association with other environment-related factors help to explain the prevalence of STH observed in this study. Finally, this study contributes for the understanding of STH distribution in a biodiverse and environmental complex area in southern Brazil.

## Funding

Marina Ziliotto received a fellowship from Coordenação de Aperfeiçoamento de Pessoal de Nível Superior - CAPES (Brazil). Joel Henrique Ellwanger receives a postdoctoral fellowship from CAPES (Programa Nacional de Pós-Doutorado – PNPD/CAPES, Brazil). José Artur Bogo Chies receives a research fellowship from Conselho Nacional de Desenvolvimento Científico e Tecnológico - CNPq (Bolsa de Produtividade em Pesquisa - Nível 1A, CNPq, Brazil) and has research project funded by CAPES (CAPES AUXPE 686/2020; Brazil).

## CRediT author statement

Marina Ziliotto: Conceptualization, Methodology, Validation, Formal analysis, Investigation, Data Curation, Writing - Original Draft, Visualization, Project administration. Joel Henrique Ellwanger: Conceptualization, Methodology, Validation, Formal analysis, Investigation, Data Curation, Writing - Review & Editing, Visualization, Project administration, Supervision. José Artur Bogo Chies: Investigation, Writing - Review & Editing, Supervision, Funding acquisition.

## Conflicts of interest

The authors declare no conflicts of interest.
