# Supplementary material for: Soil-transmitted helminths detected from environmental samples in a campus of southern Brazil
Source: Sci One Health. 2023 May 10;1:100016. doi: 10.1016/j.soh.2023.100016 (PMC11262280; doi:10.1016/j.soh.2023.100016)
Supplement: Multimedia component 1 [file mmc1.pdf]

Ziliotto, Ellwanger e Chies (2023)

Formulário para avaliação socioambiental e de atividade antrópica

Data:_________ Local de coleta:____

Coordenadas geográficas:______________________________ / ______________________________

Número da amostra de solo:___________ Aplicador:__________________________

Condições climáticas (referentes ao dia e local/cidade de aplicação do formulário)

Temperatura mínima:_____ºC Temperatura máxima: _____ºC Temperatura média: _____ºC

Tempo: ( ) ensolarado ( ) nublado ( ) chuvoso

Informações e instruções básicas para preenchimento do formulário:

- O enfoque deste formulário está em estudos parasitológicos, mas o mesmo pode ser útil para estudos de outras disciplinas.
- O formulário tem valor qualitativo (auxilia no registro de informações básicas sobre o local de estudo).
- Assinalar apenas uma opção para cada um dos 17 itens do formulário (escolher a opção que melhor representa o local de estudo no momento da aplicação do formulário).
- Preencher o formulário no local onde as coletas de dados/amostras serão realizadas, considerando como área limite o alcance de visão do aplicador (em todas as direções).
- Assinalar as opções com base em todas as informações disponíveis sobre o local de estudo no momento de preenchimento do formulário (informações visuais, faladas, escritas, etc.).

Formulário

ITEM 1 - Zoneamento:
( ) Zona urbana - área privada/domiciliar
( ) Zona urbana - área de circulação pública (ruas, pontos de ônibus, becos, universidades, etc.)
( ) Zona urbana - parques, praças ou similares
( ) Zona com atividade rural/agricultural
( ) Zona fora do perímetro urbano e sem atividade rural/humana
( ) Unidades de conservação, áreas protegidas, etc.

ITEM 2 - Tipo de solo:
( ) Arenoso
( ) Argiloso
( ) Humoso/orgânico
( ) Outro - Especificar:_______________________________

ITEM 3 - Condição do solo:
( ) Seco
( ) Úmido
( ) Encharcado

ITEM 4 - Vegetação do local:
( ) Naturalmente ausente
( ) Ausente em decorrência de ação humana (desmatamento, concretagem, mineração, etc.)
( ) Vegetação ornamental (predominante) - considerar aqui também a vegetação de praças, jardins, parques e similares
( ) Gramínea/arbustiva natural
( ) Gramínea/arbustiva natural degradada
( ) Mata (arbórea) preservada
( ) Mata (arbórea) degradada/artificial/reflorestada

ITEM 5 - Habitações/edificações humanas:
( ) Regulares de padrão alto, médio ou popular
( ) Irregulares/precárias (chão de terra nas casas, ruas não pavimentadas, sem saneamento, etc.)
( ) Ausência de habitações/edificações humanas

ITEM 6 - Poluição sonora (sons em intensidade excessiva):
( ) Ausente/sons leves
( ) Presente
( ) Presente de forma intensa
( ) Não se aplica

ITEM 7 - Saneamento ambiental (rede de esgoto/água tratada):
( ) Necessário em decorrência de habitação/atividade humana no local e presente de forma adequada
( ) Necessário em decorrência de habitação/atividade humana no local, mas ausente ou presente de forma inadequada/insuficiente
( ) Sem necessidade de saneamento ambiental/não se aplica

ITEM 8 - Descarte de esgoto doméstico no ambiente:
( ) Ausente
( ) Presente
( ) Não se aplica

ITEM 9 - Descarte de esgoto industrial no ambiente:
( ) Ausente
( ) Presente
( ) Não se aplica

ITEM 10 - Criadouros artificiais de larvas de mosquitos (com ou sem larvas vivas no momento da aplicação do questionário):
( ) Ausente
( ) Presente

ITEM 11 - Resíduos sólidos (lixo ou descartes) domésticos:
( ) Ausente ou armazenado de forma adequada (exemplo: em lixeiras)
( ) Presente

ITEM 12 - Resíduos (lixo ou descartes) industriais/biológicos oriundos de produção de média/grande escala:
( ) Ausente ou armazenado de forma adequada (exemplo: em lixeiras)
( ) Presente
( ) Não se aplica

ITEM 13 - Circulação esperada de humanos no local:
( ) Ausente
( ) Pouco frequente
( ) Frequente
( ) Intensa
( ) Não se aplica/não esperada

ITEM 14 - Animais domésticos - cães e/ou gatos (avistamento ou indicativos, como fezes ou pegadas):
( ) Ausente
( ) Presente, avistamento de animal - ( ) cães; ( ) gatos; ( ) outros:___________________
( ) Presente, fezes - ( ) cães; ( ) gatos; ( ) outros:___________________
( ) Presente, pegadas ou outros indicativos - ( ) cães; ( ) gatos; ( ) outros:___________________

ITEM 15 - Animais sinantrópicos - especialmente pombos e/ou roedores (avistamento ou indicativos, como fezes ou pegadas):
( ) Ausente
( ) Presente, avistamento de animal - ( ) pombos; ( ) roedores; ( ) outros:___________________
( ) Presente, fezes - ( ) pombos; ( ) roedores; ( ) outros:___________________
( ) Presente, pegadas ou outros indicativos - ( ) pombos; ( ) roedores; ( ) outros:___________________

ITEM 16 - Animais de criação - gado, suínos, equinos, galinhas/aves domésticas, entre outros (avistamento ou indicativos, como fezes ou pegadas):
( ) Ausente
( ) Presente, avistamento de animal - ( ) gado; ( ) suínos; ( ) equinos; ( ) aves domésticas; ( ) outros:___________________
( ) Presente, fezes - ( ) gado; ( ) suínos; ( ) equinos; ( ) aves domésticas; ( ) outros:___________________
( ) Presente, pegadas ou outros indicativos - ( ) gado; ( ) suínos; ( ) equinos; ( ) aves domésticas; ( ) outros:___________________

ITEM 17 - Fezes humanas no local de coleta ou proximidades:
( ) Ausente
( ) Presente
